# Changes in Couple Relationship Dynamics among Low-Income Parents in a Relationship Education Program Are Associated with Decreases in Their Children’s Mental Health Symptoms

**DOI:** 10.3390/children5070090

**Published:** 2018-06-30

**Authors:** Emma Sterrett-Hong, Becky Antle, Brianna Nalley, Monica Adams

**Affiliations:** Kent School of Social Work, University of Louisville, Louisville, KY 40292, USA; emma.sterrett@louisville.edu (E.S.-H.); becky.antle@louisville.edu (B.A.); brianna.nalley@louisville.edu (B.N.)

**Keywords:** prevention, mental health, interpersonal violence, parenting

## Abstract

Witnessing intimate partner violence (IPV) among parents negatively impacts millions of children in the United States each year. Low-income families are disproportionately affected by IPV compared to middle- and high-income individuals, and are beginning to be the focus of IPV secondary prevention interventions, including relationship education programs. Despite these developments, few studies have examined changes in psychosocial functioning among children of parents participating in relationship education programs. The current study examined the extent to which changes in specific couple dynamics among individuals from low-income backgrounds participating in a couple relationship education program, Within My Reach, were associated with changes in child mental health symptoms. A second purpose of this paper is to examine whether changes in parent–child relationship quality mediates the association between changes in couple dynamics and changes in child mental health difficulties. Participants (*n* = 347) were parents who participated in Within My Reach as part of programming offered at a large community agency. Decreases in negative couple conflict behaviors, including conflict engagement, withdrawal and compliance, over the course of the program were linked to decreases in child mental health difficulties. In addition, increases in parent–child relationship quality partially mediated the associations between decreases in compliance, as well as increase in overall couple relationship quality, and decreases in child symptoms. Community-based couple relationship education programs for low-income families can potentially have multiple positive impacts throughout the family system, including for children.

## 1. Introduction

Intimate partner violence (IPV) is a widespread social problem with far-reaching consequences for adults and children. Close to 18% of children in the United States have been exposed to IPV in their lifetime [1]. Currently, a little over eight million children in the United States live in homes with IPV [2]. There are significant costs associated with this violence. Adults and children living in homes with IPV experience numerous physiological and psychological outcomes, including low birth weight babies and miscarriage, depression, anxiety and chemical dependency disorders, and delays in cognitive and emotional development [3,4,5,6].

The rate of IPV is higher among low-income populations than among women from other socio-economic backgrounds [7], and the complex interplay between poverty and IPVs results in even more negative consequences among this group than among middle- or high-income women. For example, limited financial resources worsens mental health problems arising from IPV, such as post-traumatic stress disorder [8]. Additionally, IPV can lead to women remaining in poverty as forced isolation, shame and mental and physical health problems can make it difficult to obtain or maintain employment [9]. Likewise, limited financial resources can make it more difficult to obtain safe and secure housing which is also related to a higher risk of IPV, perhaps due to limited ability to escape abuse or to increased stress which can lead to greater conflict [10]. Moreover, as children who witnessed IPV are more likely to experience IPV in their own adult relationships [11], and experiencing IPV as a child is associated with lower levels of educational completion [12], adult women from low-income backgrounds experiencing IPV may have more difficulty obtaining employment due to lower levels of educational attainment.

As a result of the deleterious effects of IPV on both adults and children, interventions have been developed to both intervene with IPV after it has already started occurring and to prevent it before it begins. One of the relationship education programs used as a universal prevention strategy which has the most empirical support is the Prevention and Relationship Enhancement Program (PREP) [10,11,12,13,14]. Recently, the developers of PREP have created an adapted curriculum for use with low-income individuals, called Within My Reach (WMR) [15]. WMR’s curriculum is focused on low-income individuals who may or may not be in a romantic relationship [16,17]. Because WMR is meant for individuals versus a couple, it allows for freedom to discuss and further expand on possibly triggering or upsetting safety issues that could occur within a relationship—such as violence [18]. The goal of WMR is for participants to explore what a healthy relationship is versus a toxic one, as well as how romantic relationships impact their children, their careers and themselves. Because this program has been created for lower-income individuals, it also purposefully addresses any specific difficulties related to financial struggles [18]. Participants in WMR have reported high levels of satisfaction with the program and increased knowledge of healthy relationship dynamics [17,18,19]. In addition, participants of WMR have reported decreases in distress within their relationships [19] and a significant decrease in withdrawal when faced with conflicts within their romantic relationships [18]. A robust literature has demonstrated the link between negative inter-parental dynamics, including those addressed in relationship education programs to prevent IPV, and child psychosocial functioning [20,21]. Witnessing conflict between parents is linked to increased emotional difficulties, behavior problems, and social problems among children [22,23,24,25,26]. However, very few studies have examined changes in child mental health symptoms among children whose parents are involved in relationship education interventions aimed at increasing healthy relationship behaviors and preventing IPV. A study comparing the impact of a couple-focused intervention program to a parenting intervention program indicated that children benefited from both the couple-focused and parenting intervention [27]. Additionally, the couple-focused intervention enhanced the quality of the romantic relationships for mothers, which led to reduced mother-report of child behavior problems; however, in fathers, reduced dysfunctional parenting mediated the association between participation in the intervention and reduced child behavior problems [27]. These findings suggest that associations between changes in couple functioning and changes in child adjustment among parents involved in relationship education programs, as well as the extent to which those associations are mediated by changes in parent–child interactions, are worthy of additional empirical examination.

Several theories have been utilized to provide guidance to help explain the links between negative inter-parental dynamics, including negative conflict resolution behaviors and relationship dissatisfaction, and child mental health difficulties. In general, these literatures can be divided into theories that describe a direct effect of negative couple dynamics on child mental health through internal mental and emotional processes that occur within the child (e.g., social learning theory [28], attachment theory [29], cognitive-contextual theory [30]), and those that describe negative inter-parental dynamics affecting child mental health through their effects on other aspects of family functioning, such as parent–child relationship quality (e.g., emotional spillover effects theory [31], family systems theory [32]). For this paper, we will utilize two specific theories from these traditions as guides, social learning theory and emotional spillover effect theory, although they are not mutually exclusive to each other or other related theories explaining the links between couple functioning and child mental health. According to Social Learning Theory, children look to adults in their environment for examples or models for how to behave in social environments [28]. According to this theory, there are two ways in which children might be affected by their parents’ negative inter-parental interactions, imitation and definitions [33]. Imitation refers to a child imitating a parent using physical force to get what they want from a partner. Definitions refers to a child learning and developing certain values based on what they observe from how their parents resolve and engage in conflict; in other words, if a child sees one parent using physical violence against the other to get what they want, then a child might develop an ethical value that it is acceptable to hurt people [33]. Furthermore, according to this theory something referred to as “vicarious reinforcement” can occur. A child might observe a certain behavior exhibited by their parent, such as the use of physical violence, and then see the consequences of that violence, which might be that the other parent retreats and does not engage in further conflict [28]. This, in turn, may increase the likelihood that children may engage in such behaviors.

A second theory, the Emotional Spillover Effect Theory, suggests that the effect of having a healthy couple relationship “spills over” on parent–child relationships, making them healthier as well [31,34]. In other words, when parents have a healthy, functioning relationship those healthy aspects will likely transfer over into how they relate to their children or vice versa [31]. Likewise, if healthier parenting relationships create a healthier parent–child relationship then it can be expected that negative or unhealthy parenting relationships might have negative effects on a parent–child relationship. Most often, witnessing or being involved in parental conflict makes it more likely for children to externalize or internalize problems (often associated with behavior) as well as have a negative impact on a child’s overall adjustment [31,32,35,36]. Since more positive parent–child relationships are associated with fewer child mental health difficulties [37], the link between healthier couple interactions and fewer child mental health symptoms may be mediated by improvements in parent–child relationship quality.

Importantly, despite the strongly supported links between inter-parental conflict and child psychosocial difficulties, very few studies have examined whether improvements in couple functioning occurring over the course of a relationship education intervention will be associated with changes in child mental health symptoms. The current study represents secondary data analysis of data from the Within My Reach project in Louisville, Kentucky. Main study findings have been previously reported elsewhere, including that participants in the WMR project evidenced significant decreases in physical and emotional abuse, as well as isolation behaviors [38]. This study seeks to examine whether changes in one parent’s report of relationship satisfaction and conflict resolution behaviors among parents participating in a relationship education program are linked to changes in child mental health problems, and the extent to which those associations are direct as highlighted by Social Learning Theory, and the extent to which they are mediated by changes in Parent–Child Relationship Quality, consistent with the Emotional Spillover Effect Theory. Of note, the purpose of the WMR intervention was to help prevent IPV and improve knowledge of healthy relationship behaviors, and therefore, we expect that there will be positive changes in couple relationship dynamics. However, as this was a community and not a clinical sample, we do not necessarily expect large changes in relationship behaviors at the group level, since partners who already have healthy relationships may not evidence large changes in their behavior after relationship education [39,40]. Still, we expect that, to the extent there are improvements in couple relationship dynamics for particular parents, those improvements will be associated with improvements in child mental health, consistent with studies finding significant associations between levels of change in relational and individual outcomes when there is variability in change across participants, despite modest or no group mean change in a variable [41,42]. In addition, as effects of couple relationship education on parent–child relationship quality and child behaviors are less well-studied areas of empirical research, whether there will be changes in these domains is considered an exploratory examination.

The research questions in this study are:

1. Are changes in conflict-related behaviors and relationship quality associated with changes in child mental health symptoms among children of parents involved in a relationship education preventive intervention?
2. Do changes in parent–child relationship quality mediate the association between changes in couple dynamics and child mental health symptoms?

## 2. Materials and Methods

### 2.1. Design

This study was conducted with a convenience sample of clients who attended a relationship education program, Within My Reach, as part of a grant to expand the programming at a large community agency, which offers co-located healthcare and social services in Louisville, Kentucky. The 15-h Within My Reach curriculum was delivered once a week for four weeks, four hours each week, except the last session which was three hours of session content plus an hour to complete measures. Retention from beginning to end of the curriculum was high with 92% of the participants completing all four weeks [38]. Facilitators of the intervention participated in a three-day on-site training, as well as quarterly supervision. Fidelity to the manual was monitored and found to represent 90–100% across all sessions [43]. Approval was obtained from the University of Louisville Institutional Review Board, study # 188.07, on 2 May 2007. The pre-test was administered on the first day of the Within My Reach workshop prior to the start of programming. The post-test was administered on the last day of class.

### 2.2. Sample

The analytic sample for the current study included 347 adults who enrolled in the Within My Reach intervention, were parents, and completed supplementary measures related to their children and parent–child relationship and completed measures at both pre-test and post-test. Participants were recruited through printed advertisements, participant referral, and invitation by social service agency staff. The majority of the participants, or 88%, were female. In addition, the sample was racially and ethnically diverse, with 67% of the sample being African American, 26% being White, 2.3% being Multiracial, 1.4% being Native American, 1.4% being Other, 0.6% being Hispanic/Latino, and 0.3% being Asian American/Pacific Islander. The participants in WMR, as intended by the manual created by the intervention developers, participated individually without their partners. They reported on all measures for the study.

### 2.3. Measures

Participant demographics measured included gender, age, race, religion, relationship status, receipt of various social services, education, occupation, employment status, income and number of children.

#### 2.3.1. Relationship Dynamics-Conflict Behaviors

Participants completed the Conflict Resolution Styles Inventory (CRI) [44] at the start of the Within My Reach program directly after program completion. At post-test, participants were asked to report on the same relationship on which they reported at pre-test. The CRI has evidenced good face validity, evidence for convergent validity, and evidence for concurrent and predictive criterion-related validity [45,46]. The Conflict Resolution scale comprised four sub-scales, Conflict Engagement, Withdrawal, Compliance and Positive Conflict Resolution. Cronbach’s alphas for the subscales in the current study ranged from 0.70 to 0.80. Change in conflict-related behaviors was calculated by subtracting the post-test score from each subscale from the pre-test score for that subscale.

*Relationship Dynamics-Relationship satisfaction and quality* were measured pre- and post-intervention using a seven-item version of the Dyadic Adjustment Scale [47]. The Dyadic Adjustment Scale (DAS) [47] assesses the quality of couple relationship by examining four areas: satisfaction, cohesion, consensus, affection and expression. It has been used in both research and clinical settings. It has been shown to discriminate between distressed and non-distressed individuals as well as married and divorced couples. Construct validity has been reported as 0.88 and 0.86. The Cronbach’s alpha for the DAS in the current study was 0.85. Change in DAS was calculated by subtracting the DAS score at pre-test from the DAS score at post-test.

*Parent–Child Relationship Quality* Parent–child relationship quality was measured using 15 items from the Involvement, Communication and Satisfaction with Parenting subscales of the Parent–Child Relationship Inventory (PCRI) [48], which measures parent–child relationship quality and emotions experienced by participants in their role as parents. Parents were asked to complete the PCRI in reference to their child, and if they had more than one child, to complete the questionnaire in reference to their child they considered to be most challenging. Internal consistency, test-retest, and convergence validity for these PCRI subtests has been established [49]. The Cronbach’s alpha of the scale created for this study is 0.74. Change scores in parent–child relationship quality were calculated by subtracting the pre-test PCRI score from the post-test score.

#### 2.3.2. Child Mental Health Symptoms

Child mental health symptoms were measured using the Pediatric Symptom Checklist-17 (PSC-17) [50]. The PSC-17 is a shorter form of the 35-item Pediatric Symptom Checklist [51] and has demonstrated good construct and criterion validity [52]. The PSC-17 is a screening measure of general mental health in children. Cronbach’s alpha in the current sample was 0.91.

### 2.4. Data Analysis

**Figure 1:**
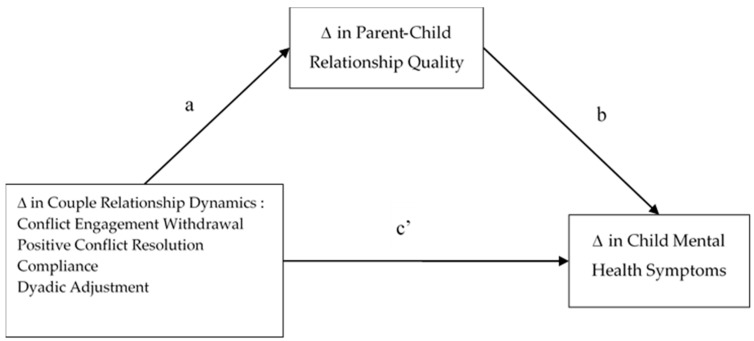
Conceptual Model Depicting Parent–Child Relationship Quality as a Mediator of the Associations between Change in Couple Relationship Dynamics and Change in Child Mental Health Symptoms.

We conducted bivariate correlations to examine bivariate associations between change in the predictors, change in couple relationship dynamics; the proposed mediator, parent–child relationship quality; and the outcome variable, change in child mental health symptoms. To examine whether change in parent–child relationship quality mediated the associations between change in couple relationship quality and change in child symptoms, we conducted analyses using the *process* mediation macro in SPSS, which utilizes bootstrapping to generate a sampling distribution of the indirect effect in ordinary least squares regression-based path analysis to produce confidence intervals for mediation effects [53]. We specified 10,000 boot-strapping iterations. The conceptual model used for the mediation analyses is displayed in Figure 1.

## 3. Results

### 3.1. Descriptive Statistics

Descriptive statistics of demographic variables and major study variables are in Table 1. As participants were allowed to skip items, 79% of participants had complete data. For all variables, if participants reported on at least one item in a scale, missing responses were replaced by zeros and included in the calculation of their scale scores so that their data and power could be retained. Among participants who had missing data, the percentage of missing data imputed ranged from 2.5% to 13%. The pre-test scores were roughly in the middle of the possible range for the couple conflict behavior variables and dyadic adjustment scales.

We conducted *t*-tests to examine differences between mean pre-test scores and mean post-test scores. Statistically significant differences between pre-test and post-test scores were observed for conflict engagement, *t* = 2.68, *p* < 0.01; withdrawal, *t* = 4.23, *p* < 0.01; positive resolution, *t* = 2.21, *p* < 0.05; parent–child relationship quality, *t* = −4.11, *p* < 0.01; and child mental health symptoms *t* = 2.60, *p* < 0.01. In addition to conducting *t*-tests of pre-post-test mean differences, we also calculated reliable change scores, which allow for examinations of whether mean change over time at the group level is large enough to distinguish it from measurement error [54]. None of the pre-post mean change scores represent reliable change at the group level. However, of note, the Pediatric Symptoms Checklist has a suggested clinical cutoff of 15 when using response options of 0 to 2 [50]. Of the 132 children who were above the clinical cutoff for mental health symptoms at pre-test, 32% or 24%, were no longer above the clinical cutoff at post-test.

Although 92% of the sample was retained from the pre-test to the post-test, we also examined differences in regard to both demographic (i.e., sex, race, socio-economic status) variables and base-line scores on measures between participants who completed all four sessions of the WMR program and those that did not. There were no significant demographic differences or differences with regard to scores on the measures, with the exception that a higher percentage of other ethnic minorities, 12%, did not complete the training compared to African American, 2%, and White, 2%, participants.

### 3.2. Bivariate Correlations

We conducted correlation analyses to examine bivariate associations between major study variables, including change in couple functioning and change in parent–child relationship quality, and the outcome, change in child mental health symptoms (see Table 2). Decreases in the three unhealthy couple relationship dynamics, conflict engagement, withdrawal, and compliance, were significantly associated with decreases in child mental health symptoms, although only two of those associations were significant after a Bonferroni correction [55] for multiple comparisons. The two healthy relationship variables, overall dyadic adjustment and positive conflict styles, were not associated with change in child symptoms at the bivariate level. Change in the hypothesized mediator variable, parent–child relationship quality, was significantly negatively associated with change in child mental health symptoms, such that an increase in parent–child relationship quality was associated with a decrease in child symptoms.

### 3.3. Multiple Linear Regression

We conducted multiple linear regression to examine associations between changes in the various relationship dynamics studied and changes in child mental health symptoms in the same model. Improvements in parent–child relationship quality were significantly associated with decreases in child mental health symptoms, β = −0.18, *p* < 0.01, even after a Bonferroni correction for multiple comparisons (see Table 3)*.* In addition, decreases in conflict engagement were significantly associated with decreases in child symptoms, β = 0.18, *p* < 0.01, although it was no longer significant after the Bonferroni correction. There were no other statistically significant associations in the full multiple linear regression model.

### 3.4. Mediation Analyses

Next, we examined mediation by utilizing the SPSS *process* macro [53] to determine whether change in parent–child relationship quality would mediate associations between the five couple relationship dynamics (i.e., conflict engagement, positive conflict style, withdrawal, compliance and overall dyadic adjustment) each and change in child mental health symptoms. We estimated mediation models even in which the predictor and outcome variable were not correlated at the bivariate level or in the multivariate analyses, consistent with recent literature that an indirect effect can exist, even if there is not a bivariate correlation between the predictor and outcome variable [56].

Results of the mediation analyses are presented in Table 4. With regard to specific parent conflict-related behaviors, changes in conflict engagement, and withdrawal, were significantly associated with changes in child mental health symptoms. Specifically decreases in conflict engagement, β = 0.28, *p* < 0.01; and withdrawal, β = 0.25, *p* < 0.01; were associated with decreases in child symptoms. Changes in compliance and positive conflict resolution were not associated with changes in child mental health symptoms. In addition, changes in all four conflict-related behaviors were significantly associated with changes in parent–child relationship quality, with decreases in negative behaviors and increases in positive conflict resolution being associated with increases in parent–child relationship quality. Parent–child relationship quality partially mediated the association between decreases in compliance and decreases in child behavior problems, with an indirect effect of 0.04 and the confidence interval spanning from 0.01 to 0.08 (see Table 3). Parent–child relationship quality did not mediate the associations between the other conflict-related behaviors and child mental health symptoms.

In terms of overall couple relationship quality, increase in dyadic adjustment was not associated with a decrease in child mental health symptoms in the full model. In contrast, an increase in overall relationship satisfaction was associated with increases in parent–child relationship quality. Mediation analyses indicated increases in parent–child relationship quality partially mediated the association between increases in dyadic adjustment and decreases in child mental health problems. The mediation effect was −0.02 and confidence interval −0.05 to −0.01.

## 4. Discussion

In this study, decreases in negative conflict behaviors among adults from low-income backgrounds participating in a relationship education program, Within My Reach (WMR), were associated with decreases in child mental health symptoms. In addition, changes in parent–child relationship quality acted as a mediator in the associations between changes in compliance and changes in child mental health symptoms, as well as between changes in overall relationship satisfaction and changes in child symptoms. On the other hand, changes in parent–child relationship quality did not mediate the other associations between changes in couple dynamics and change in child symptoms.

This study sought to examine the extent to which associations between changes in couple dynamics and changes in child mental health symptoms would be both direct consistent with Social Learning Theory, and mediated by changes in parent–child relationship quality, consistent with theories that highlight the inter-connectedness of family climate and relationships, such as Emotional Spillover Effect Theory. In this study, decreases in child mental health symptoms associated with decreases in negative inter-parental conflict among low-income adults in a relationship education class were, to some extent, independent of effects of parent–child relationship quality. While these findings are preliminary, it could be the case that a decrease in inter-parental conflict styles results in there being less modeling of negative coping with conflict to which children are exposed, as suggested by Social Learning Theory [28,57]. Such an explanation would be consistent with a social learning view that one cause of child misbehavior is witnessing negative behavior by parents which serves as a model for ways to interact with others.

Importantly, parent–child relationship quality was a partial mediator of the effect of one partner-report of two couple relationship dynamics, although the mediation effects were modest. Changes in parent–child relationship quality partly explains the association between changes in overall couple relationship quality and child mental health symptoms. It is possible that improvements in the couple relationship leads to less stress for parents, who then are able to have a more positive relationship with their children. This idea is consistent with literature demonstrating links between healthy couple functioning and lower levels of stress for partners [58,59], and studies demonstrating a link between lower levels of parental stress and higher levels of parent–child relationship quality [60,61]. The other couple relationship dynamic whose effect was partially mediated was compliance. It could be the case that parents who developed and expressed more assertiveness in the couple relationship generalized that assertiveness to their relationships with their children. Parental self-competence and assertiveness in interactions with their children has been found to be associated with fewer child mental health symptoms [62,63,64]. However, these findings would need to be replicated before confidence could be more fully placed in these conclusions.

This study also demonstrates the potential for changes in participant relationship functioning to have cascading effects. Importantly, although there were statistically significant changes in couple dynamics, parent–child relationship quality, and child mental health symptoms from pre-test to post-test, there were no pre-post changes in variables at the group mean level that represent reliable change that is significantly not affected by measurement error. However, findings suggest preliminarily that for those participants in an IPV prevention program who do evidence change at the couple level, there may be corresponding changes in child mental health symptoms. Consistent with general family systems theory which emphasizes mutual causality and the inter-dependence of individuals and sub-systems in a larger system [65], this study underscores the inter-connected nature of family relationships and how changes in one sub-system can be related to changes in individual well-being for other members of the system. It is also noteworthy that 24% of children rated as being in the clinical range on a mental health symptoms screener before the intervention were reported as being in the normal range after the intervention. While this is a promising finding, adding more direct instruction in effective parenting techniques to relationship education programs may be necessary to see a greater reduction in the percent of children who screen positive for a clinical level of mental health symptoms. Additionally, increases in positive resolution strategies were associated with increases in parent–child relationship quality, although not with changes in child mental health symptoms in the multivariate models. Therefore, additional supports besides an increase in positive behaviors, such as a decrease in negative couple behaviors, may be needed to decrease child mental health symptoms. This study possessed several strengths. The study included an ethnically diverse sample, which increases generalizability of findings. In addition, the study focused on low-income individuals, who have been the subject of fewer relationship education studies. The study also examined a relatively novel domain of change related to participation in a relationship education program: changes in child mental health symptomatology. Finally, this was a community-based study, in which client participation on on-going services at a large community agency were examined, increasing the external validity of the study.

There were also limitations to this study. The relationship behaviors were measured using self-report, which can be biased. In addition, the same reporter, one parent, reported on both their behaviors and their children’s symptoms, which could have led to common method variance. Future research should incorporate assessment from other reporters, particularly the other parent, or include observations to strengthen confidence in study findings. Furthermore, although enrollment of just one partner in IPV intervention and prevention programs is common and can lead to changes in relationships as reported by the participant involved [66,67], the lack of report regarding relationship dynamics or child functioning from the other partner/parent limits interpretations that can be drawn regarding the extent to which changes actually occurred. In addition, since child symptoms were not the original purpose of this research project, there was limited information available about the children reported on, such as their age or sex. This paper focused on the uni-directional association between changes in couple dynamics and changes in child mental health symptoms, we acknowledge the influence that child symptoms have both on parent–child relationship quality and couple dynamics. Therefore, future research should examine the bi-directional nature of these associations. Finally, this study did not include a control group and only included participants who had post-test data, and thus, findings can only be generalized to individuals completing the WMR program in community settings.

In conclusion, this study contributes to the literature by examining how participants in an intimate partner violence prevention program who demonstrate changes in their romantic relationships may also see changes in their relationship with their children and their children’s mental health. Change in healthy relationship behaviors over the course of a relationship education program were associated with decreases in child mental health difficulties. The WMR project targets healthy relationship behaviors and educates parents on the importance of their relationships on child well-being, in contrast with the traditional approaches to violence prevention that utilize individual, pathology-oriented content. Thus, for adults who evidence increases in healthy relationship behaviors with their partners they may see changes in a variety of areas of family life, suggesting the potentially wide-reaching effects that increased policy and funding investments in preventive relationship programming could have for low-income families.

## Figures and Tables

**Table 1:** Descriptive Statistics of Demographic and Major Study Variables.

	*m* or *n* (%)	*SD*	Possible Range	Pre-Post Diff. *t*-Test Score	Reliable Change Score ^t^
Race					
African-American	234 (67%)				
White	90 (26%)				
Multiracial	8 (2.3%)				
Native American	5 (1.4%)				
Hispanic	2 (0.6%)				
Other	6 (1.7%)				
Did not respond	4 (1%)				
Age	34	10.46			
Sex					
Female	305 (87%)				
Male	42 (12%)				
Did not respond	2 (1%)				
Annual Family Income					
<$10,000	184 (53%)				
$10,000–$20,000	75 (21%)				
$20,000–$30,000	36 (10%)				
>$30,000	21 (6%)				
Didn’t respond	33 (10%)				
Employment Status					
Full-time	56 (16%)				
Part-time	36 (10%)				
Unemployed	209 (60%)				
Other	40 (11%)				
Did not respond	8 (2%)				
Education					
≤8th grade	14 (4%)				
Some high school	46 (13%)				
12th grade or General Education Diploma	129 (37%)				
Some college or Technical School	146 (42%)				
Bachelors or Graduate degree	9 (3%)				
Pre-test in Conflict Engagement		10.16	3.82	1–20	
Pre-test in Withdrawal		11.13	3.64	1–20	
Pre-test in Positive Conflict		12.26	3.48	1–20	
Pre-test in Compliance		9.04	3.34	1–20	
Pre-test in Dyadic Adjustment Scale	19.95	7.38	0–36		
Pre-test in Parent–Child Relationship	45.15	6.64	1–60		
Pre-test in Child Problem Behaviors	27.84	7.24	1–51		
Pre-Post Δ in Conflict Engagement	−0.48	3.4		2.61 **	−0.24
Pre-Post Δ in Withdrawal	−0.84	3.6		4.23 **	−0.36
Pre-Post Δ in Positive Conflict Resolution	0.34	3.1		−2.21 *	0.12
Pre-Post Δ in Compliance	−0.26	3.4		1.30	−0.08
Pre-Post Δ in DAS	0.75	6.12		−1.88 ^a^	0.13
Pre-Post Δ in Parent–Child Relationship	1.2	5.2		−4.11 **	0.26
Pre-Post Δ in Child Problem Behaviors	−0.73	4.9		2.60 **	−0.28

^a^ < 0.10, * < 0.05, ** < 0.01, ^t^ = A reliable change score must exceed 1.96 to represent psychometrically sound change.

**Table 2:** Bivariate Correlations between Major Study Variables.

	1.	2.	3.	4.	5.	6.	7.	8.	9.
1. Ethnicity	—								
2. Age	−0.02	—							
3. Family Income	−0.09	0.11 *	—						
4. Δ Conflict Engagement	−0.09	0.02	0.01	—					
5. Δ Positive Resolution	−0.03	0.03	−0.06	−0.29 **^,t^	—				
6. Δ Withdrawal	−0.02	0.08	−0.06	0.36 **^,t^	−0.09	—			
7. Δ Compliance	0.02	−0.04	−0.02	0.24 **^,t^	0.01	0.29 **^,t^	—		
8. Δ Dyadic Adjustment	0.00	0.05	0.11 *	−0.24 **^,t^	0.22 **^,t^	−0.24 **^,t^	−0.17 **	—	
9. Δ Parent–Child Relationship	−0.02	−0.02	0.01	−0.14 *	0.05	−0.08	−0.13 *	0.14 *	—
10. Δ Child Symptoms	−0.10 ^a^	0.05	0.01	0.22 *	−0.05	0.19 **^,t^	0.11 *	−0.08	−0.23 **^,t^

^a^ < 0.10, * < 0.05, ** < 0.01, ^t^ = significant after Bonferroni correction.

**Table 3:** Results of Multiple Regression Analyses Examining Predictors of Change in Child Mental Health Symptoms.

Predictors:	β	*t*	*p*
∆ Parent–Child Relationship Quality	−0.20	−3.51	0.00 **^,t^
∆ Conflict Engagement	0.16	2.56	0.01 *
∆ Withdrawal	0.12	1.92	0.06 ^a^
∆ Positive Resolution	0.02	0.36	0.72
∆ Compliance	−0.01	−0.17	0.86
∆ Dyadic Adjustment	0.04	−0.10	0.91

^a^ < 0.10, * < 0.05, ** < 0.01, ^t^ = significant after Bonferroni correction.

**Table 4:** Results of Mediation Analyses Examining Parent–Child Relationship Quality as a Mediator of Associations between Change in Couple Dynamics and Change in Child Mental Health Symptoms.

	Mediator as Outcome: ∆ Parent–Child Relationship Quality			Outcome: ∆ Child Mental Health Symptoms			Indirect Effect		
Predictors:	Coeff.	*SE*	*p*	Coeff.	*SE*	*p*	Effect	Lower Limit Confidence Interval	Upper Limit Confidence Level
∆ Conflict Engagement	−0.18	0.08	0.02	0.28	0.04	<0.001 ^t^	0.03	0.00	0.07
∆ Parent–Child RQ	—	—	—	−0.18	0.07	<0.001 ^t^			
∆ Withdrawal	−0.18	0.07	0.02	0.25	0.06	<0.001 ^t^	0.03	0.00	0.07
∆ Parent–Child RQ	—	—	—	−0.18	0.04	<0.001 ^t^			
∆ Positive Resolution	0.20	0.08	0.01	−0.09	0.04	0.21	−0.04	−0.08	0.00
∆ Parent–Child RQ	—	—	—	−0.19	0.07	<0.001 ^t^			
∆ Compliance	−0.22	0.07	0.01	0.11	0.07	0.11	0.04	0.01	0.08
∆ Parent–Child RQ	—	—	—	−0.19	0.04	<0.001 ^t^			
∆ Dyadic Adjustment	0.14	0.04	<0.01	−0.05	0.04	0.21	−0.02	−0.05	−0.01
∆ Parent–Child RQ	—	—	—	−0.19	0.04	<0.001 ^t^			

^t^ = significant after Bonferroni correction.
